# A highly efficient genetic transformation system for broccoli and subcellular localization

**DOI:** 10.3389/fpls.2023.1091588

**Published:** 2023-03-02

**Authors:** Yongyu Zhao, Dongxu Yang, Yumei Liu, Fengqing Han, Zhansheng Li

**Affiliations:** Key Laboratory of Biology and Genetic Improvement of Horticultural Crops, Ministry of Agriculture, Institute of Vegetables and Flowers - Chinese Academy of Agricultural Sciences, Beijing, China

**Keywords:** broccoli, agrobacterium, CRISPR/Cas9, subcellular localization, genetic transformation, transgenic

## Abstract

**Introduction:**

Agrobacterium-mediated genetic transformation has been widely used for the identification of functional genes and regulatory and developmental mechanisms in plants. However, there are still some problems of low genetic transformation efficiency and high genotype dependence in cruciferous crops.

**Methods:**

In this study, broccoli, a worldwide Brassica crop, was used to investigate the effects of genotype, explant type, concentration of hygromycin B used during seedling selection, overexpression vector type, RNAi and CRISPR/cas9 on the genetic transformation efficiency. At the same time, two vectors, PHG-031350 and PHG-CRa, were used for subcellular localization of the glucoraphanin synthesis-related gene FMOGS-OX5 and clubroot resistance gene by a PEG-Ca2+-mediated transient transformation system for broccoli protoplasts. Finally, the Agrobacterium-mediated genetic transformation system of broccoli was optimized and improved.

**Results and Discussion:**

This study showed that hypocotyl explants are more suitable for Agrobacterium-mediated transgene and CRISPR/Cas9 gene editing of broccoli. In contrast to previous studies, we found that 5 mg/L hygromycin B was more advantageous for the selection of resistant broccoli sprouts, and genotype 19B42 reached the highest transformation rate of 26.96%, which is higher than that in Brassica oleracea crops. In addition, the inbred line 19B42 successfully achieved high genetic transformation of overexpression, RNAi and CRISPR/Cas9 vectors; thus, it is powerful recipient material for the genetic transformation of broccoli. Subcellular localization proved that the glucoraphanin metabolism-related gene Bol031350 and clubroot resistance gene CRa were both expressed in the cytoplasm and nucleus, which provided a scientific basis for studying the regulation of glucosinolate metabolism and clubroot resistance in cruciferous crops. Therefore, these findings will provide new insight into the improvement of the genetic transformation and molecular breeding of Brassica oleracea crops.

## Introduction

Broccoli (*Brassica oleracea* L. var *italica*) is an internationally popular vegetable, and the planting area of broccoli in China has recently increased yearly, exceeding 86,000 hm^2^ in 2019 (Huang et al., 2021a). Broccoli is known to be rich in vitamin C, proteins, and minerals and contain the anticancer active ingredient sulforaphane, which can significantly reduce the risk of a variety of cancers, cardiovascular and cerebrovascular diseases, Alzheimer’s disease, myopia, and depression (Fahey et al., 2002; Li et al., 2017; Bessler and Djaldetti, 2018; Li et al., 2019a; Li et al., 2021b).

At present, genetic engineering has played an important role in gene functional analysis, and it can also help breeders quickly obtain target plant traits with homozygous genetic backgrounds, greatly shortening breeding times. To date, plant genetic transformation methods generally include the Agrobacterium-mediated method, pollen tube pathway, particle bombardment, nanomaterial-mediated transient transformation, and virus-induced gene silencing (VIGS) (Men et al., 2003; Kant and Dasgupta, 2017; Wang et al., 2019; Lv et al., 2020; Li et al., 2022). Agrobacterium-mediated genetic transformation has the characteristics of universality, wide applicability and simple operation, so it has been widely used in plants. In 1983, herbicide-resistant transgenic tobacco was first obtained using Agrobacterium-mediated genetic transformation (Liu et al., 2018).

The Agrobacterium-mediated genetic transformation system has been widely used in wheat, rice, maize, tomato, rape and other crops (Thomzik, 1995; Hu et al., 2003; Li and Li, 2003; Sharma et al., 2009; Bates et al., 2017; Zhong et al., 2018; Van Eck et al., 2019). Based on this method, gene silencing, overexpression and gene editing have been applied in plants, and a number of new germplasm resources have been improved for resistance to stress (cold resistance, salt resistance, bolting resistance, etc.) disease (blight, rust, black rot, virus disease), insects (*Helicoverpa armigera*, diamondback moth, cabbage caterpillar, flea beetle), and herbicides (glyphosate, acetochlor, fomesafen), as well as high nutrition (vitamins, proteins, phytic acid, γ-aminobutyric acid). Regulatory genes related to plant development have been identified and analyzed, including high yield (*IPA1*) and lodging resistance (*OsTCP15*) in rice (Zhang et al., 2017; Banerjee and Roychoudhury, 2018), plant height and yield traits in maize (Luo et al., 2022; Wang et al., 2022a), pod setting and branching in soybean (Guan et al., 2015; Shim et al., 2019; Su et al., 2022), tomato flavor traits (Tikunov et al., 2020), and bitterness regulation in cucumber (Shang et al., 2014). For most cruciferous crops, a complete genetic transformation system has been established; However, for a few crop species, these systems have not yet been perfected. Agrobacterium-mediated transformation and CRISPR/Cas9 gene editing have been widely applies to rape (Probsting et al., 2020), cabbage (*cry1Ia8*, *cry1C*) (Yi et al., 2016; Yi et al., 2017), and broccoli (*Cry1Ac*) (Cao et al., 2002). However, research on transformation system of Chinese cabbage is lagging behind the other Brassica crops, such as rapeseeds, cabbage and so on, so VIGS must be used to identify functional genes (Murata and Orton, 1987). In 2020, genetic transformation of Chinese cabbage mediated by Agrobacterium was performed (Zhao et al., 2021), and the CRISPR/Cas9 gene editing system will provide important support for the functional gene identification of Chinese cabbage in the future. At the same time, the genetic transformation system mediated by Agrobacterium has shown great differences among different species, and there is obvious genotype differences. Therefore, genotype is one of the important factors affecting plant genetic transformation. In addition, previous studies found that the explant type, concentration of hygromycin B used during seedling selection and vector type also affect the genetic transformation efficiency in different plants. Most studies have shown that in cruciferous crops, hypocotyls are generally better than cotyledons as explants (Zhao et al., 2021), the concentration of hygromycin B used during seedling selection differs across varieties, and the selection concentration of rape is often higher than that of diploid cabbage, broccoli, cauliflower, and kale (Odell et al., 1985; Liu et al., 2008; Kumar and Srivastava, 2016). It has been reported that the efficiency of gene editing is often lower than that of overexpression and gene silencing, and there are generally no significant differences between overexpression and gene silencing results (Htwe et al., 2014; Ali et al., 2016).

At present, research on the genetic transformation of broccoli has mainly focused on growth and development, nutritional regulation and resistance (Li et al., 2019b; Han et al., 2021; Huang et al., 2021b). *FMO* genes and MYB transcription factors related to glucosinolate secondary metabolites in broccoli can affect the synthesis of glucosinolates (Gigolashvili et al., 2007; Wang et al., 2017; Neequaye et al., 2021; Kim et al., 2022) and positively regulate the expression of the storage-related gene *ClpB1* (Wang et al., 2022b). For broccoli, an efficient genetic transformation system is very important. Clubroot caused by *Plasmodiophora brassicae* is a major disease of Brassica crops worldwide, and it usually occurs on rapeseed, cauliflower, broccoli, Brussels sprouts, Chinese cabbage, and radish. To avoid this disease, breeders must introduce clubroot resistance (CR) genes from the European turnip into susceptible crop lines. The *CRa* gene encoding a TIR-NBS-LRR protein has been shown to confer specific resistance to the clubroot pathogen *Plasmodiophora brassicae* (Xie et al., 2022).

In this study, we optimized a highly efficient Agrobacterium-mediated transformation system by testing 5 different broccoli genotypes, 5 vectors, including overexpression, RNAi and CRISPR/Cas9, 2 explant types, and 4 gradient concentrations of hygromycin B for seedling selection. Our findings greatly improve the efficiency of the broccoli genetic transformation system and provide a scientific basis for the establishment of efficient transgenic and gene editing technology systems widely used in other cruciferous crops.

## Materials and methods

### Plant materials

**Figure 1:**
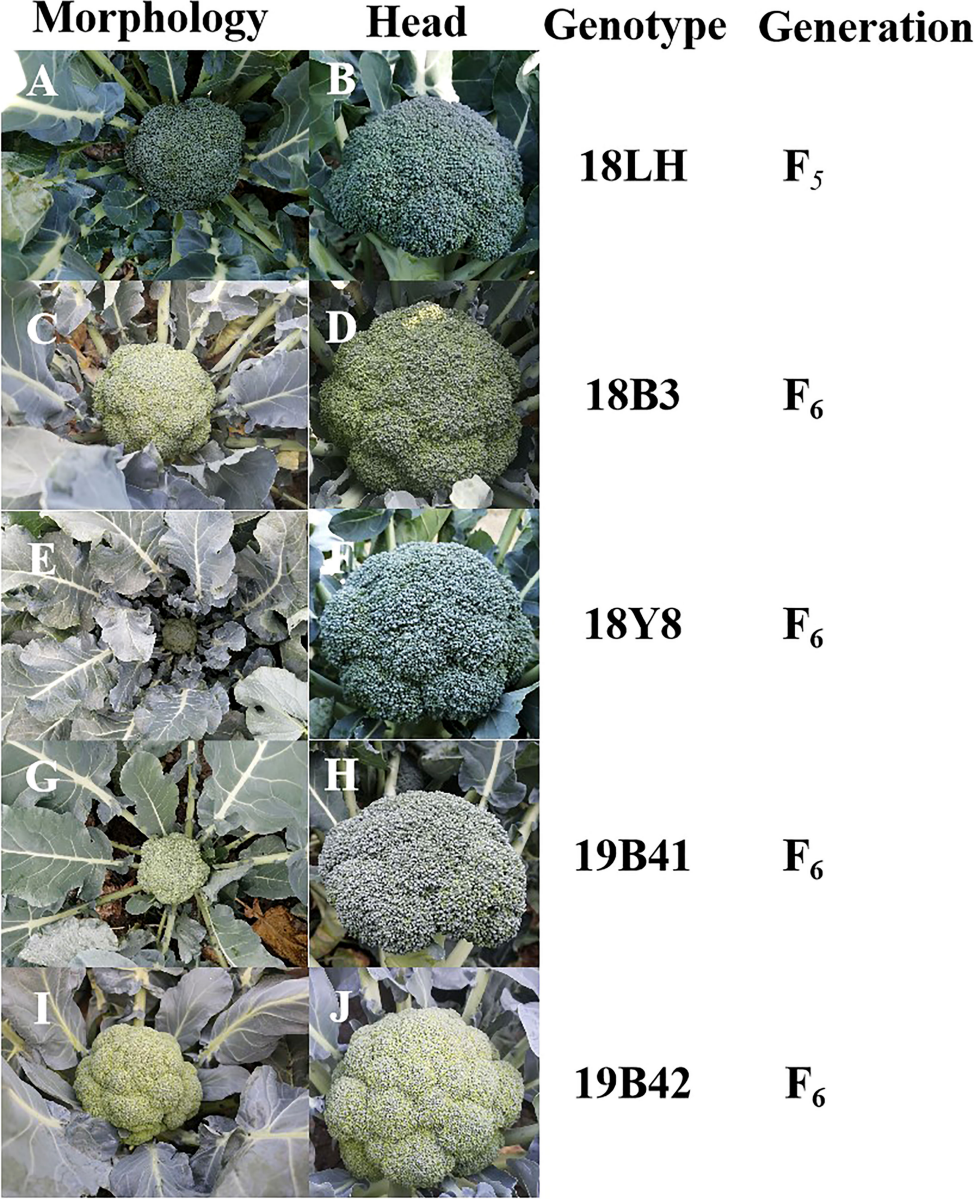
The genetic information and profiles of 5 broccoli inbred lines. **(A, B)** Morphology and head image of 18LH genotype broccoli plant. **(C, D)** Morphology and head image of 18B3 genotype broccoli plant. **(E, F)** Morphology and head image of 18Y8 genotype broccoli plant. **(G, H)** Morphology and head image of 19B41 genotype broccoli plant. **(I, J)** Head and plant profiles image of 19B42 genotype broccoli plant.

The materials used in this experiment were 5 broccoli inbred lines: 18LH, 18Y8, 18B3, 19B41, and 19B42 (**Figure 1**). All the materials were cultivated by the Institute of Vegetables and Flowers, Chinese Academy of Agricultural Sciences (IVF-CAAS). This study was carried out from April 2021 to September 2022.

All the materials were planted and identified in autumn 2021 (**Figure 1**). 18LH was an inbred line at the F_5_ generation, and it showed an early maturing, semi-open plant type, a solid stem with few lateral branches (1-3), and a dome-shaped head with thin buds. 18Y8 was an inbred line at the F_6_ generation, and it showed a middle-late maturing, semierect plant type, a solid stem with no lateral branches, and a dome-shaped head with medium buds. 18B3 was an inbred line at the F_6_ generation, and it showed an early maturing, erect plant type, a solid stem with many lateral branches (4-6), and a dome-shaped head with medium buds. 19B41 was an inbred line at the F_6_ generation, and it showed an early maturing, erect plant type, a solid stem with few lateral branches (1-2), and a semidome-shaped head with medium buds. 19B42 was an inbred line at the F_6_ generation, and it showed a middle-early maturing, semierect plant type, a solid stem with no lateral branches (1-2), and a dome-shaped head with thin buds.

### Plasmid vectors

**Figure 2:**
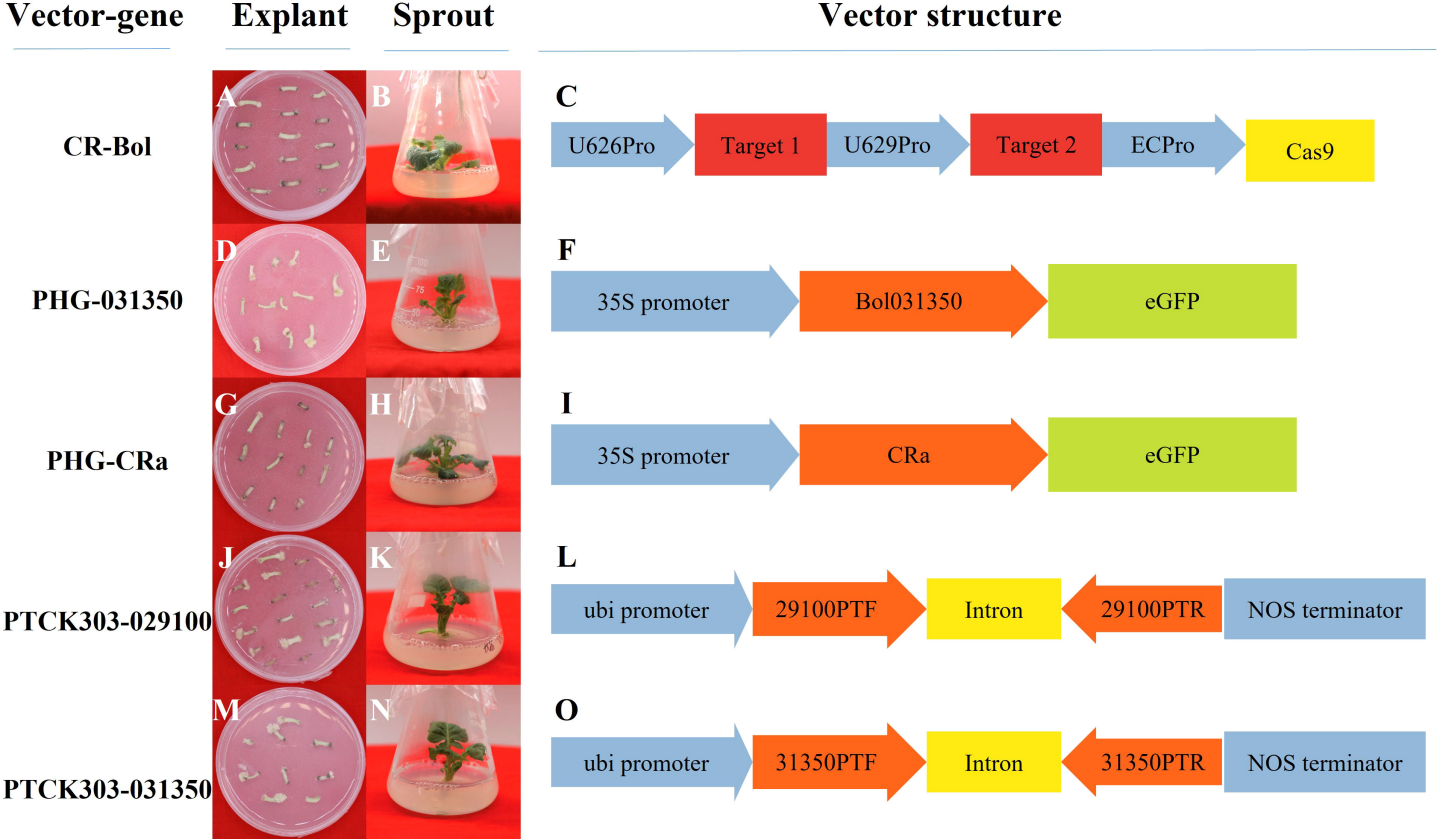
Profiles of the vector performance in 19B42 and their structural differences. **(A-C)** Hypocotyls and sprouts from19B42 with vector CR-*Bol* accompanied, and the structure of vector CR-*Bol*.**(D-F)** Hypocotyls and sprouts from19B42 with vector PHG-*031350* accompanied, and the structure of vector PHG-*031350*. **(G-I)** Hypocotyls and sprouts from19B42 with vector PHG-*CRa* accompanied,and the structure of vector PHG-*CRa*. **(J-L)** Hypocotyls and sprouts from19B42 with vector PTCK303-*029100* accompanied,and the structure of vector PTCK303-*029100*.**(M-O)** Hypocotyls and sprouts from19B42 with vector PTCK303-*031350* accompanied,and the structure of vector PTCK303-*031350*.

In total, 5 plasmid vectors were designed and constructed in our study (**Figure 2**): the overexpression vectors PHG-*031350* and PHG-*CRa*, the RNAi vectors PTCK303-*029100* and pTCK303-*031350*, and the gene editing vector CRISPR/Cas9-*Bol* (CR-*Bol*).

### Sterilization of seeds and the acquisition of explants

Broccoli seeds were selected and placed in a 50 mL centrifuge tube, soaked in 75% alcohol for 3 min and then in 8% sodium hypochlorite for 8 min, and finally washed 3 times with sterile water for 3 min. After drying on sterile filter paper, the seeds were placed on MS medium for 5-7 days. Under sterile conditions, the hypocotyls of broccoli seedlings were cut into 8-10 mm sections.

### Transformation of agrobacterium and preparation of agrobacterium solution

Agrobacterium (GV3101) stored at -80°C were partially thawed at room temperature, and a total of 0.5 μg of plasmid DNA was added to each 100 μL of bacterial solution. After mixing, the solution was placed on ice for 5 min and in liquid nitrogen for 5 min, followed by a water bath at 37°C for 5 min and on ice for 5 min. Then, 700 μL of antibiotic-free liquid lysogeny broth (LB) was added and incubated for 2-3 h at 28°C under shaking conditions. After centrifugation at 6000 rpm for 1 min, the supernatant was removed. Approximately 100 μL of supernatant was used to resuspend the Agrobacterium, which was evenly coated on solid LB containing the appropriate antibiotics and inverted at 28°C for 36-48 h. Finally, 1 mL of liquid LB containing the appropriate antibiotics was placed in a 1.5 mL centrifuge tube, and each colony was inoculated into the centrifuge tube, shaken at 28°C for 12-15 h and stored at 4°C.

The day before infection, 200 μL of bacterial solution was spread on solid LB containing the appropriate antibiotics and then cultured at 28°C for 24 h. During the infection, the Agrobacterium was resuspended in liquid MS medium, the concentration was adjusted to an absorbance of 0.4-0.6 at 600 nm, and an appropriate amount of acetosyringone was added for later use.

The configuration of each medium in the experiment is shown in **Table 1**.

**Table 1:** The medium composition and nutritional components used in this experiment.

MS Medium	M519(g/L)	Sucrose(g/L)	Agar(g/L)	6-Benzylaminopurine (mg/L)	1-Naphthylacetic acid (mg/L)	Acetosyringone (mM)	Timentin (mM)	Hygromycin B (mg/L)
Nutritional components								
Seeding medium	4.43	28	8	0	0	0	0	0
Premedium	4.43	28	8	1	0.1	0	0	0
Coculture	4.43	28	8	1	0.1	1	0	0
Delay medium	4.43	28	8	1	0.1	0	3	0
Selection medium	4.43	28	8	1	0.1	0	3	8-6-5-4
Growth medium	4.43	28	8	0.2	0.1	0	3	0

### Infection and subsequent culture of explants

The explants were cultured in the preculture medium for 2 days and then transferred to a sterile petri dish. Infection solution was added, the explants were soaked for 10 min, and the excess infection solution was poured out. The explants were placed on sterile filter paper to dry and then placed on coculture medium for 36-48 h in the dark.

After 36-48 h in the dark, the explants were transferred to delayed medium for 4-5 days and then transferred to selection medium with different concentrations of hygromycin B for 14 days, which was repeated three times (n=3). After the resistant sprouts grew, they were transferred to growth medium.

### DNA extraction and PCR identification

Genomic DNA was extracted from broccoli sprouts using the modified CTAB method (Chaparro-Encinas et al., 2020). The specific primer pair P1 (HYG-F: GCTTCTGCGGGCGATTTGTGT; HYG-R: GGTCGCGGAGGCTATGGATGC) was designed using Primer3 software (California, USA) online (http://primer3.ut.ee/), and all the transgenic plants were individually amplified and identified.

The PCR program was as follows: predenaturation at 94°C for 3 min; 35 cycles of denaturation at 94°C for 30 s, annealing at 62°C for 45 s, and extension at 72°C for 1 min; final extension at 72°C for 7 min; and storage at 4°C. The PCR results were detected by 1.2% agarose gel electrophoresis.

### Subcellular localization

The overexpression vectors PHG-*CRa* and PHG-*031350* with enhanced green fluorescent protein (EGFP) tags were transiently transformed into broccoli protoplasts by a PEG-Ca^2+^-mediated method, which was carried out by Yang’s report (Yang et al., 2022). Then, temporary glass slides were made for observation and imaging based on the methods reported by Domozych and Sant (Domozych et al., 2020; Sant'Ana et al., 2020).

### Statistical analysis of the data

Statistical analysis was performed with SPSS 22 software (IBM, Chicago, USA), and the data are presented as the mean ± S.D. (n=3). One-way ANOVA with Tukey’s test and Student’s t test was used to determine the different factors influencing the differentiation rate and genetic transformation efficiency. The differentiation rate (%) was calculated as the number of explants with adventitious buds in the total number of explants (× 100). The genetic transformation efficiency (%) was calculated as the number of positive plants in the total number of explants (× 100).

## Results

### Effect of hygromycin B concentration on the differentiation rate

**Figure 3:**
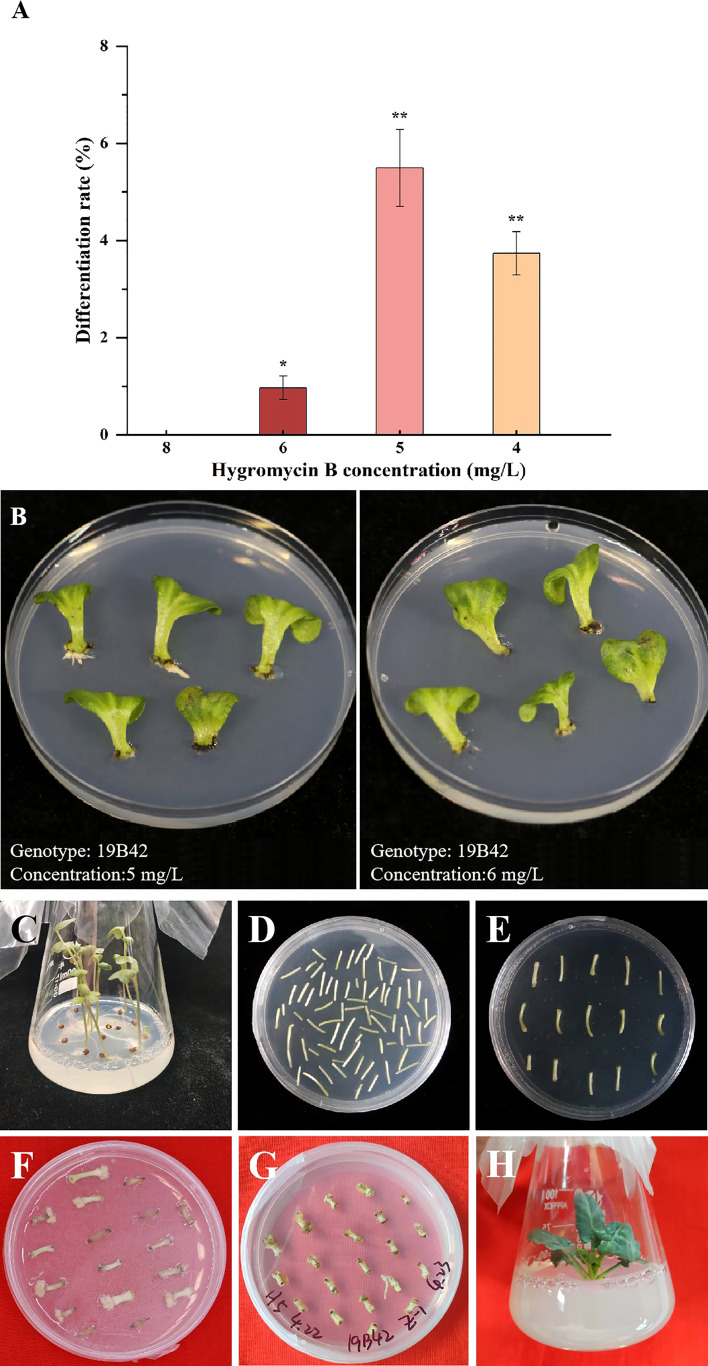
The differentiation rate of genotype 19B42 under hygromycin B, and its explants performance. **(A)** Statistical significance levels are as follows: ns indicates no significant difference at a level of 0.05, * indicates a significant difference at p<0.05; and ** indicates a significant difference at p<0.01. **(B)** Performance of cotyledons (19B42) after 45 days of selective culture. **(C-H)** Performance of hypocotyls(19B42) in transformation events.

Direct selection of hygromycin-B-resistant transformant was carried out in our experiment, and 4 gradient concentrations were set (4 mg/L, 5 mg/L, 6 mg/L, and 8 mg/L). We found that there were significant differences in the differentiation rate among the four gradients concentrations (*p*<0.05) (**Figure 3**). No callus occurred when the concentration was 8 mg/L, so the differentiation rate of all materials was 0. When the selection concentration decreased to 6 mg/L, some explants began to differentiate into adventitious sprouts with a differentiation rate of 0.96% detected in 19B42. When the concentration was further reduced to 5 mg/L, the differentiation rate of all materials ranged from 0.8% to 26.92%, and the genotype with the lowest rate was 18Y8 (0.8%), while that with the highest rate was 19B42 (26.92%). When the selection concentration was reduced to 4 mg/L, the highest differentiation rate declined to 3.90% (19B42), and the differentiation rate ranged from 3.23% (18B3) to 3.90% (19B42). At the same time, the explants were induced to differentiate into calli and adventitious roots.

### Effect of genotype on the differentiation rate of adventitious buds

**Figure 4:**
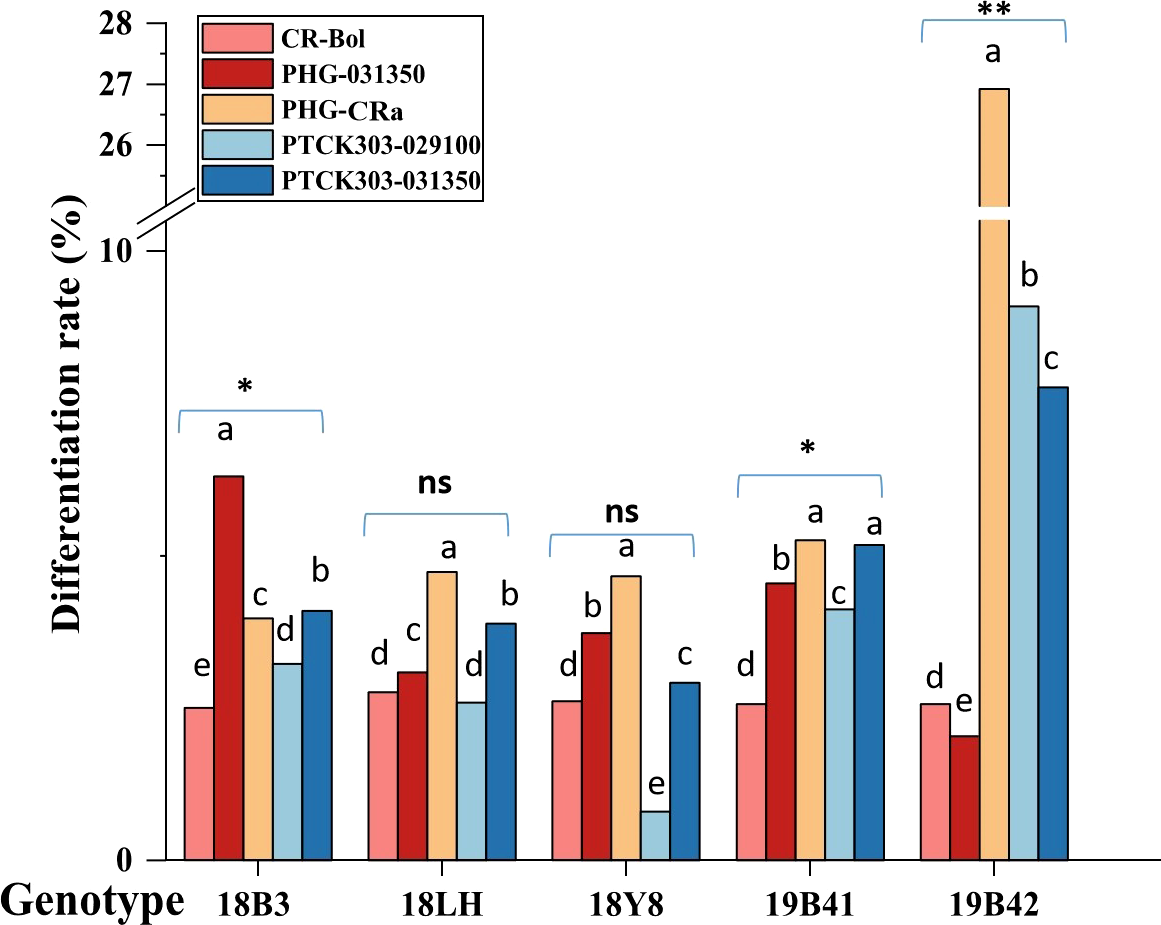
The differentiation rate of 5 vectors detected in different broccoli genotypes. Statistical significance levels are as follows: ns indicates no significant difference at a level of 0.05, * indicates a significant difference at p<0.05; and ** indicates a significant difference at p<0.01. Different lowercase letters indicate significant differences at p<0.05.

**Figure 4** shows that the differentiation rate of 5 different broccoli genotypes varied greatly when the concentration of hygromycin B was 5 mg/L, and the differentiation rates of different vectors were also detected in different genotypes. The differentiation rates of the 5 vectors in inbred line 19B42 ranged from 2.03% to 26.92% with an average value of 9.67%, among which the overexpression vector PHG-*CRa* showed the highest differentiation rate of 26.92%. This was followed by the RNAi vectors PTCK303-*029100* and PTCK303-*031350* with differentiation rates of 9.09% and 7.77%, respectively. The overexpression vector PHG-*031350* had the lowest differentiation rate of 2.03%, followed by the gene editing vector CR-*Bol* at 2.56%. **Figure 2** shows the different vectors and their structures. When the transferred vector was PHG-*CRa*, the broccoli inbred line 19B42 showed the highest differentiation rate of 26.92%, which was higher than that of genotypes 19B41 (5.26%) and 18LH (4.73%) (**Figure 4**). For vector PHG-*031350*, the differentiation rates of broccoli genotypes ranged from 2.03% (19B42) to 6.31% (18B3), with an average value of 3.68%. When the RNAi vector PTCK303-*029100* was transferred into the 5 genotypes, as shown in **Figure 4**, there was a significant difference in the differentiation rates, ranging from 9.09% and 0.8%. The same difference was also observed for the RNAi interference vector PTCK303-*031350*, with differentiation rates of 7.77% and 2.91% for 19B42 and 18Y8, respectively.

### Effect of explant type on the differentiation rate

Two different types of explants, hypocotyls and cotyledons, were selected for our study. When hypocotyls were selected as explants, the different vector types also affected the differentiation rate of the plants among the 5 different genotypes of broccoli. Differentiation rates ranged from 2.50% to 2.76% for the gene editing vector CR-*Bol*, while they ranged from 3.97% to 26.92% for the overexpression vector PHG-*CRa* and from 2.03% to 6.31% for the overexpression vector PHG-*031350*. The differentiation rates of the RNAi vectors PTCK303-*029100* and PTCK303-*031350* were 0.8% - 9.09% and 2.91% - 7.77%, respectively.

In our study, when cotyledons were selected as explants, none of the vectors were effective in inducing adventitious sprouts, and some genotypes could induce calli or differentiate into adventitious roots (**Figure 3**). The differentiated sprouts were all obtained from hypocotyls, and most of the cotyledons formed calli or differentiated into adventitious roots and could not be effectively differentiated to obtain resistant sprouts.

### Identification of genetically transformed T0 plants

**Figure 5:**
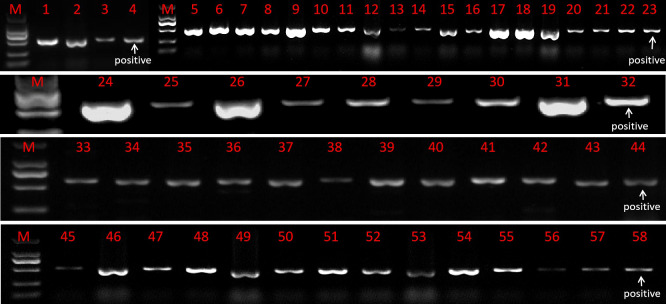
The identification results of some genetically transformed strains. “M” is the DL2000 DNA marker. Numbers 4, 23, 32, 44, and 58 were all positive controls. Numbers 1 to 3 represent the CR-*Bol* gene detected in 19B42, numbers 5-22 represent the PTCK303-*031350* gene detected in 19B42, numbers 24-31 represent the PHG-*CRa* gene detected in 18LH, numbers 33-40 represent the PTCK303-*029100* gene detected in 19B42, numbers 41-43 represent the PTCK303-*029100* gene detected in 18Y8, numbers 45-50 represent the PHG-*031350* gene detected in 19B42, and numbers 51-57 represent the PHG-*031350* gene detected in 18B3.

**Figure 6:**
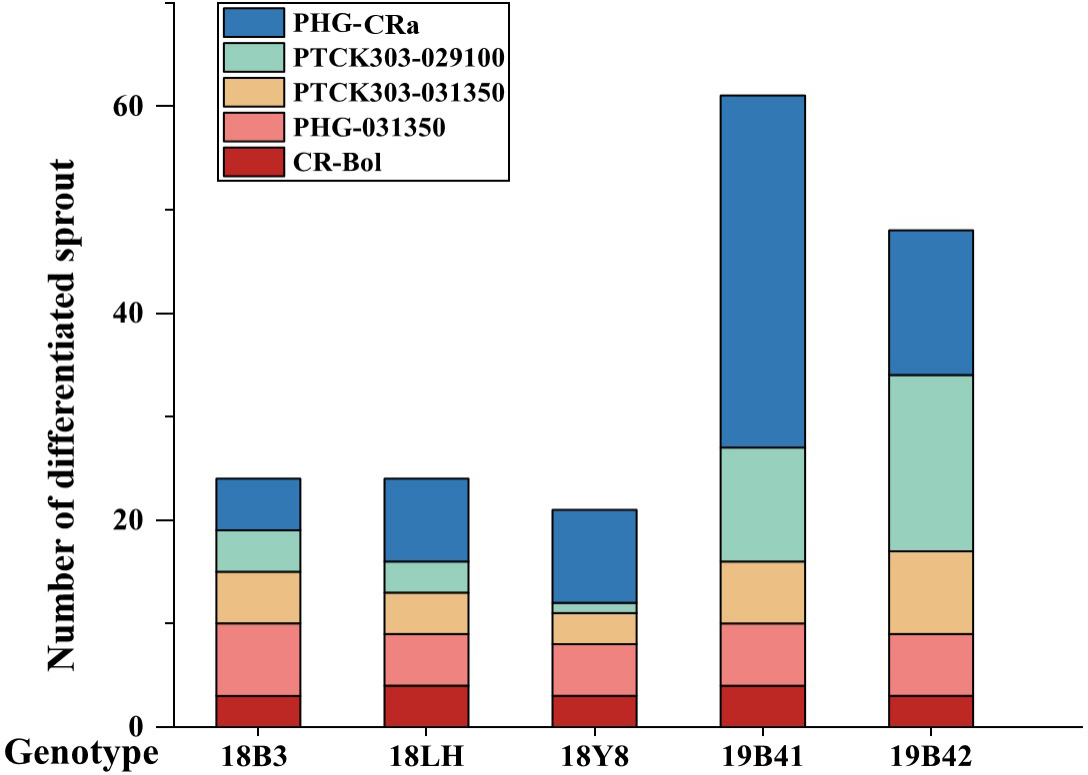
The difference in differentiated sprout numbers among 5 broccoli genotypes.

The resistant sprouts were subjected to PCR amplification using the hygromycin B-specific primer pair P1, and 178 plants had specific bands (**Figure 5**). Ultimately, a total of 178 transformed T0 broccoli plants were obtained in this study, including 24 plants from genotype 18LH, of which 4 were obtained for CR-*Bol*, 5 were obtained for PHG-*031350*, 8 were obtained for PHG-*CRa*, 3 were obtained for PTCK303-*029100*, and 4 were obtained for PTCK303-*031350*; 21 plants were obtained from genotype1 8Y8, of which 3 were obtained for CR-*Bol*, 5 obtained for PHG-*031350*, 9 were obtained for PHG-*CRa*, 1 was obtained for PTCK303-*029100*, and 3 were obtained for PTCK303-*031350*; 24 plants were obtained from 18B3, of which 3 were obtained for CR-*Bol*, 7 were obtained for PHG-031350, 5 were obtained for PHG-*CRa*, 4 were obtained for PTCK303-*029100*, and 5 were obtained for PTCK303-031350; 61 plants were obtained from 19B41, of which 4 were obtained for CR-*Bol*, 6 were obtained for PHG-*031350*, 34 were obtained for PHG-*CRa*, 11 were obtained for PTCK303-*029100*, and 6 were obtained forPTCK303-*031350*; and 48 plants were obtained from 19B42, of which 3 were obtained for CR-*Bol*, 6 were obtained for PHG-*031350*, 14 were obtained for PHG-*CRa*, 17 were obtained for PTCK303-*029100*, and 8 were obtained for PTCK303-*031350* (**Figure 6**). **Figures 5**, **6** clearly show the number of differentiated sprouts and genetically transformed strains.

### Subcellular localization

**Figure 7:**
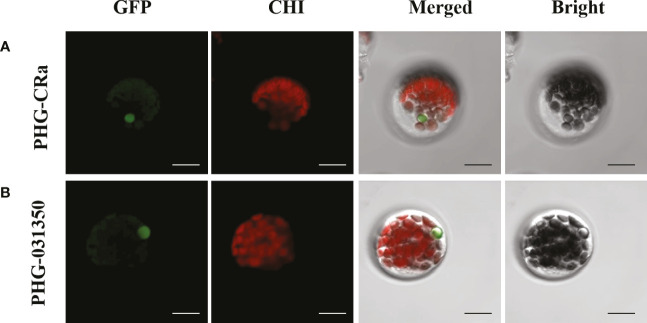
Subcellular localization of *CRa* and *Bol031350* in broccoli. **(A, B)** presented PHG-*CRa*, and PHG-*031350* were all located in the cytoplasm and nucleus and transiently co-expressed in broccoli 19B42 protoplasts; Individual and merged images of GFP and chlorophyll autofluorescence (Chl) as well as bright field images of protoplasts were shown. Scale bars = 5 μm.

Two overexpression GFP-labeled vectors were transiently transformed into broccoli protoplasts, and green fluorescence was obviously observed on the cytoplasm and nucleus under laser confocal microscopy, as shown in **Figure 7**. Therefore, this result indicated that the genes *Bol031350*, which is related to glucosinolate metabolism, and *CRa*, which is related to resistance to clubroot, were both expressed in the cytoplasm and nucleus, which was consistent with previous studies (Li et al., 2021a; Abdel-Ghany et al., 2005; Bai et al., 2014).

## Discussion

Agrobacterium-mediated genetic transformation has been widely used in plants for gene function analysis and regulatory mechanism research (Nakano and Otani, 2020; Zhang et al., 2021). It has also been widely used in cruciferous vegetable crops, including rapeseed, cabbage, broccoli, and kale. However, there are still some problems with its application to Chinese cabbage. At the same time, genotype differences also limit the use of this technology in other crops, and the extremely low transformation efficiency of different genotypes resulting in a lack of transformation need to be addressed. To optimize and improve the efficiency of genetic transformation in cruciferous vegetable crops, important influencing factors, including genotype, vector type, the concentration of hygromycin B used during seedling selection and explant type, were thoroughly investigated in this study. Genotype was the most important factor for Agrobacterium-mediated genetic transformation, and the genetic vectors could directly affect genotype to improve genetic transformation efficiency. The concentration of hygromycin B resistance was the fundamental factor for obtaining resistant sprouts, and either too high or too low of a concentration could seriously reduce the transformation efficiency or increase the probability of false positives. We have found that hypocotyls are the better choice for explants during the genetic transformation of broccoli, and almost no differentiated buds appeared when cotyledons were used due to lower differentiation, consistent with most previous reports (Metz et al., 1995; Chakrabarty et al., 2002). Subcellular localization studies showed that the glucosinolate metabolism-related gene *Bol031350* and the clubroot resistance gene *CRa* were both expressed in the broccoli the cytoplasm and nucleus, and these findings will be useful for studying the regulatory mechanism of glucosinolate synthesis and clubroot resistance in cruciferous crops (Hansen et al., 2007; Li et al., 2021).

Producing plants that can overcome the genotype dependence of genetic transformation is important. In our study, broccoli genotype 19B42 showed a higher response under all five vectors across all materials, which indicates that it could be used as a powerful transformation acceptor for gene functional analysis in the future; the second-best potential genotype is the inbred line 19B41. These two important broccoli genotypes meet the prerequisites for the verification of functional genes in broccoli and other Brassica crops, which is a very useful for future work in *Brassica oleracea* crops (Ramkumar et al., 2020; Sood et al., 2020).

In our study, it was specifically proven that the concentration of hygromycin B used during seedling selection is a major factor for effective Agrobacterium-mediated genetic transformation in broccoli, as has been widely demonstrated in wheat, maize, tomato, rape, cabbage and other crops (Radchuk et al., 2000; Chen et al., 2008; Rao et al., 2016; Xu et al., 2018; Zhou et al., 2020; Krishna et al., 2021). It is clear that the optimal selection concentration of antibiotics in Agrobacterium-mediated genetic transformation will greatly ensure and improve the efficiency of positive plant generation. In our study, 5 mg/L could not only increase the differentiation rate of different broccoli genotypes but also ensure a more efficient acquisition of positive plants. We found that this concentration is generally different from that in cabbage (8 mg/L), rape (50 mg/L) and other Brassica crops, which may be correlated with the diversity of plant hormone levels in differentiated tissues (Radchuk et al., 2000; Kim et al., 2016). Therefore, it is necessary to select a suitable concentration of antibiotic for use during seedling selection to improve the efficiency of obtaining differentiated buds and positive plants.

Furthermore, the explant plays an important role in the Agrobacterium-mediated genetic transformation system. Currently, it is reported that immature embryos, mature embryos and young ears of wheat can be used as explants for genetic transformation (Hu et al., 2003). Among vegetable crops, cotyledons are popular explants for tomato and pepper (Van Eck et al., 2019; Tikunov et al., 2020), but in eggplant, the regeneration ability of hypocotyl explants is significantly higher than that of cotyledon explants (Padma and Ravishankar, 2013). Cucurbit crops, such as watermelon, muskmelon and cucumber, generally use cotyledon nodes as explants (Fang and Grumet, 1990; Choi et al., 1994; Zhao et al., 2022). For cruciferous crops, the transformation efficiency of rape stem explants was the highest, while for radishes, cotyledons were suitable explants. Both cotyledon and hypocotyl explants have been successfully reported in cabbage.

In summary, there are few reports on the optimization of the genetic transformation of broccoli. Therefore, this study conducted a comparative study on the effect of 5 broccoli genotypes, 2 explant types, 3 vector transformations, and 4 gradient concentrations of hygromycin B on genetic transformation (Ravanfar et al., 2014), and our study will provide new evidence and new insights into improving the efficiency of Agrobacterium-mediated genetic transformation in broccoli and other Brassica crops.

## Conclusion

This experiment found that the two most important factors affecting the genetic transformation of broccoli are the genotype and the concentration of hygromycin. The 5 mg/L concentration of hygromycin B was more advantageous for obtaining resistant broccoli sprouts, and cotyledon explants were not suitable for Agrobacterium-mediated genetic transformation of broccoli. In addition, genotype 19B42 reached a higher transformation rate of 26.96% similar to that in other *Brassica oleracea* crops; thus, inbred line 19B42 will provide us with powerful recipient material for the genetic transformation of broccoli and other *Brassica oleracea* crops. Subcellular localization of the glucoraphanin metabolism-related gene *Bol031350* and clubroot resistance gene *CRa* was first carried out in broccoli, and their proteins were found in the cytoplasm and nucleus, which provided a scientific basis for studying the regulation of glucosinolate metabolism and clubroot resistance in cruciferous crops.

## Data availability statement

The original contributions presented in the study are included in the article/**Supplementary Material**. Further inquiries can be directed to the corresponding author.

## Author contributions

ZY and ZL conceived the project and wrote the manuscript. ZY and YD conducted the experiment, collected the data, and helped with manuscript preparation. ZY, YD, YL, HF, and ZL analyzed the data. ZL was the project administrator and helped review and edit this manuscript. All authors contributed to the article and approved the submitted version.
