# Supplementary material for: A highly efficient genetic transformation system for broccoli and subcellular localization
Source: Front Plant Sci. 2023 Mar 2;14:1091588. doi: 10.3389/fpls.2023.1091588 (PMC10018207; doi:10.3389/fpls.2023.1091588)
Supplement: Supplementary file 1 [file DataSheet_1.docx]

**Method for the isolation and purification of protoplasts** **was performed according to Yang et al. (2022)**

Upper fully expanded broccoli cotyledons of aseptic seedlings cultured for 7-10 d were selected, spread over the bottoms of sterile 9-cm glass Petri dishes filled with CM (Table S1**)**, and incubated in the dark at 4 °C for 12 h.

After pretreatment, the CM was discarded, a scalpel was used to make a scratch approximately 1 mm long on the back of the leaf, and 10 mL of preconfigured enzyme digestion solution (1.0 % cellulase R-10, 0.1 % pectolyase Y-23, and 0.4 M mannitol, pH 5.8) was added.

The material was placed on a shaker and enzymatically digested at 25 °C in the dark for 6 h.

After enzymatic digestion, the dishes were gently shaken in a single direction to release the protoplasts. The leaf residue was removed by passage of the material through a 50.0-mm nylon membrane sieve.

The material that passed through the sieve was collected in a 10-mL round-bottom centrifuge tube. After centrifugation of this material at 700 r/min for 5 min, the supernatant was discarded, 7.0 mL of W5 (154 mM NaCl, 125.0 mM CaCl_2_, 5.0 mM KCl and 2.0 mM MES, pH 5.7) was added to the pellet, the resulting suspension was centrifuged at 700 r/min for 3 min, and the supernatant was again discarded.

The above process was repeated twice. The protoplasts were then immediately resuspended in MMG solution (0.4 M mannitol, 15.0 mM MgCl_2_ and 4.0 mM MES, pH 5.7) at a density of 1.0-2.0×10^6^ protoplasts mL^-1^ (Figure S1).

**Table S1 CM medium detailed composition.**

| **Chemicals** | **Concentration (mg/L)** |
| --- | --- |
| KNO_3_ | 190 |
| MgSO_4_ | 37 |
| CaCl_2_·2H_2_O | 44 |
| KH_2_PO_4_ | 17 |
| FeSO_4_·7H_2_O | 2.79 |
| Na_2_-EDTA | 3.73 |
| MnSO_4_·4H_2_O | 2.23 |
| H_3_BO_3_ | 0.62 |
| ZnSO_4_·7H_2_0 | 0.86 |
| NaMoO_4_·2H_2_O | 0.025 |
| CuSO_4_·5H_2_O | 0.0025 |
| KI | 0.083 |
| CoCl·6H_2_O | 0.0025 |
| Inositol | 10 |
| Nicotinic acid | 0.5 |
| Glycine | 0.2 |
| Lactium | 100 |
| pH | 5.7-5.8 |


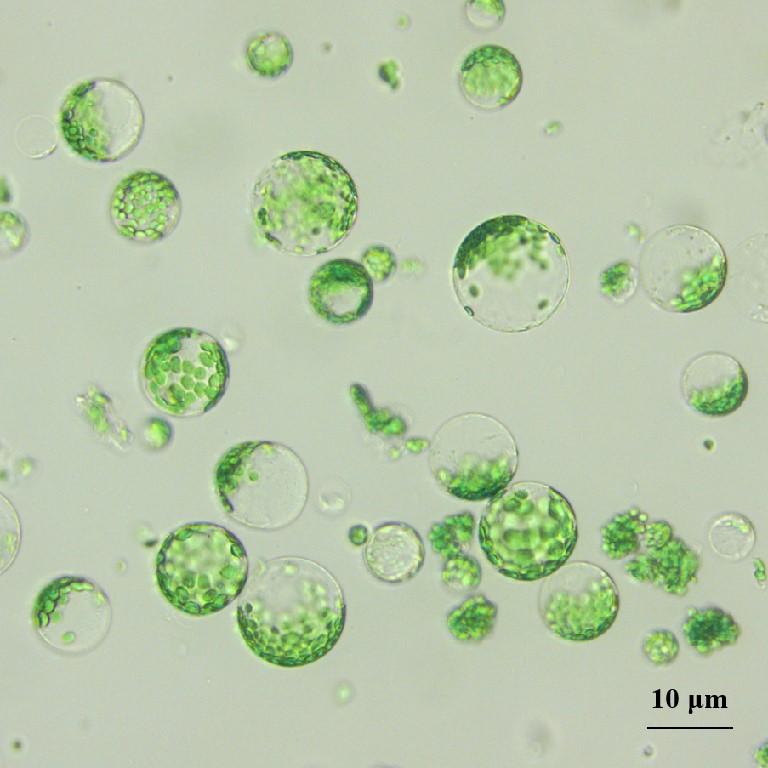


**Figure S1 protoplast of broccoli (19B42) before transformation**
